# Prevalence of sufficient MVPA among Thai adults: pooled panel data analysis from Thailand’s surveillance on physical activity 2012–2019

**DOI:** 10.1186/s12889-021-10736-6

**Published:** 2021-04-07

**Authors:** Piyawat Katewongsa, Chutima Yousomboon, Narumol Haemathulin, Niramon Rasri, Dyah Anantalia Widyastari

**Affiliations:** 1 Institute for Population and Social Research, Mahidol University, Salaya, Phutthamonthon, Nakhon Pathom, 73170 Thailand; 2 Thailand Physical Activity Knowledge Development Centre (TPAK), Institute for Population and Social Research, Mahidol University, Salaya, Phutthamonthon, Nakhon Pathom, 73170 Thailand; 3 Thai Health Promotion Foundation, 99/8 Soi Ngamduplee Thungmahamek, Sathorn, Bangkok, 10120 Thailand

**Keywords:** Physical activity, Prevalence, Thailand, Adults, Surveillance

## Abstract

**Background:**

The role of data in informing decision makers in formulating policy to improve population health is undeniably important. During the past few years, the Thai government has undertaken continuous health promotion campaigns and programs. Nevertheless, evidence of how physical activity (PA) has improved is lacking. This study aims to present PA prevalence and trends from nationally-representative surveillance data collected during 2012–2019.

**Methods:**

This study employed 8 rounds of Thailand’s Surveillance on Physical Activity (SPA) survey from 2012 to 2019 as a pooled analysis from two-panel data (SPA2012–2016 and SPA2017–2019). Multistage random sampling was applied to select Thai adults aged 18 or over to produce a nationally-representative dataset, by considering the place of residence (urban or rural), gender, and single year of age. Face-to-face interviews using a structured questionnaire were conducted in 5 regions, 13 provinces, and 36 villages to follow up 5648 individuals in Panel 1 (SPA2012–2016) and 6074 persons in Panel 2 (SPA2017–2019).

**Results:**

The prevalence (%) of Thai adults who met WHO recommendations on sufficient PA tended to increase over time, from 66.6 (CI 65–68) in SPA2012 to 70.1 (CI 69–71), 69.5 (CI 68–71), 73.1 (CI 72–74), 70.6 (CI 69–72), 73.0 (CI 72–74), 75.6 (CI 74–77), and 74.3 (73–75) in SPA2013–2019, respectively. Thai females are less physically active than males, and the prevalence of sufficient moderate and vigorous PA (MVPA) was highest among middle-aged adults (35–64 years), and lowest among older adults (65+ years). Work-related PA dominated the cumulative minutes of MVPA per week, followed by recreational PA.

**Conclusion:**

The prevalence of sufficient MVPA has fluctuated over time with a tendency to increase in the most recent years. Work-related is the most common modes of PA among Thai adults, implying further improvement in recreational physical activity is required. Workplace intervention should also be the focus in improving PA of Thai adults by encouraging their work force to engage in more occupational PA.

**Supplementary Information:**

The online version contains supplementary material available at 10.1186/s12889-021-10736-6.

## Introduction

Health-promoting physical activity (PA) is beneficial for the population of all ages. The new 2020 WHO guidelines on PA and sedentary behavior (SB) recommended an average of 150–300 min of moderate intensity or 75–150 min of vigorous intensity per week for adults to obtain the optimal benefit of PA, particularly in averting all-cause or cardiovascular-related mortality and reducing the incidence of diabetes and cancer [1]. Previous studies also found that individuals with sufficient PA have favorable mental health outcomes [2–4] and better quality of life [5, 6].

Globally, it is estimated that 28% of adults do not meet the WHO global recommendations for sufficient PA [1, 7]. The prevalence of insufficient PA varied by region and level of development of the countries, ranging from 4 to 19% in low-income countries such as Nepal, Mozambique, and Kenya, to 24–30% in lower-middle income countries such as India, Indonesia and Srilanka, and higher than 30% among upper-middle and high-income countries [8]. PA also varied by socio-economic status of individuals or families. Individuals from middle- and high-income families are more likely to engaged in recreational PA, whereas individuals from low-middle-income households are more likely to engage in transportation or work-related PA [9].

Considering its high importance, WHO has recommended PA as the best-buy policy in promoting population health [10, 11]. Policies on PA extol its value, not only for health, but also for socio-economic well-being, all of which contribute to the attainment of the Sustainable Development Goals (SDGs). Following the Bangkok Declaration on Physical Activity for Global Health and Sustainable Development 2016, the Thai government set the target that 80% of the population would be sufficiently physically active in 2020 [12]. The national strategy is also in line with the global NCD targets which include a 25% reduction in NCD-related premature mortality, and a 10% reduction in the prevalence of insufficient PA by 2025. The target was drawn from the evidence that physical inactivity was attributed to 1.3% of total Disability Adjusted Life Years (DALY) loss of Thai population [13].

Health promotion campaigns, interventions, and programs to increase PA of the Thai population have been undertaken continuously during the past few years. Nevertheless, the evidence of how PA has improved is still lacking. The existing studies on PA are mainly localized, with relatively small sample sizes that may not be generalizable. While other national surveys with large sample size are also available, there is no publication on PA prevalence since each survey has its own priority. Most published articles employing National Health Examination Surveys (NHES) focused on the prevalence and risk factors of metabolic syndrome such as hypertension, obesity, or diabetes. Sport and Exercise Surveys (2004–2011) failed to cover all PA domains, whereas the 2015 Physical Activity Survey did not use the standardized tools (i.e. GPAQ) in defining PA. In the absence of standardized measures and consistent definition of PA over times, it is difficult to obtain the best estimate of PA level of the population [14, 15].

Given the dearth of national sample data on PA prevalence of the Thai population, this study was conducted to provide estimates PA prevalence and trends from a two-panel dataset extracted from Thailand’s Surveillance of Physical Activity (SPA) 2012–19. As panel data that observes a group of population throughout a certain period of time, the results of the study should be valuable for the Thai government and policy makers, as well as for researchers who are interested in PA of the Thai population. These data should be beneficial in refining strategies and designing interventions to improve population health through PA.

## Methods

### Study design, population, and sample

This study employed data from 8 rounds of Thailand’s Surveillance on Physical Activity (SPA) from 2012 to 2019 as a pooled analysis from a two-panel dataset (SPA2012–2016 and SPA2017–2019). Designed as a longitudinal study, SPA contains a wealth of information at the individual level, particularly on PA, sedentary behavior, and socio-economic characteristics of the Thai population. Data on special issues related to emerging trends in population health and health promotion were also collected in certain rounds of the SPA.

Multistage random sampling was applied to select Thai adults aged 18 or over from a nationally-representative sample by considering the variance in geographical area (region and urban/rural), gender and age. Face-to-face interviews were conducted in 5 regions, 13 provinces, and 36 villages as repeated measures to follow up 5648 individuals in Panel 1 (SPA2012–2016) and 6074 persons in Panel 2 (SPA2017–2019). The two panels were independent sample, but driven from an identical sampling frame and sampling technique to ensure national representativeness and enable data pooling.

As a longitudinal study with panel-data design, a low attrition rate is an important indicator to ensure the quality of data. The follow-up rate of SPA in Panel 1 was remarkably high throughout the five rounds; 75.0% in SPA2012–2013, 85.2% in SPA2013–2014, 86.5% in SPA2014–2015, and 89.1% in SPA2015–2016. Similarly, the follow up rate in Panel 2 was also considerably high, with an average of 80.7% (80.7% in SPA2017–2018, and 80.6% in SPA2018–2019). Loss to follow-up was mostly due to two reasons: 1) Moved out from the village; or 2) Died. For any case that was lost to follow-up, a person with matched characteristics (gender, age group, occupation) and who lived in the same community was substituted.

### Measurement

PA was measured by using the Global Physical Activity Questionnaire (GPAQ) v.2 (Thai version) and expressed as the prevalence of sufficient MVPA. Sufficient MVPA was defined following WHO recommendation of 75-min of vigorous activity, or 150-min of moderate activity, or a combination of the two [16]. The value was calculated from weekly cumulative minutes of MVPA summed up from work-related and recreational PA at moderate and vigorous intensity, and cumulative minutes PA for transportation.

The instrument itself (GPAQ v.2-Thai version) has undergone a validity test in 2003, by involving 832 samples in the actual study sites, and using an activity tracker (Feel-fit accelerometer) developed by Biomedical Engineering Department, Faculty of Engineer, Mahidol University. As the standard measure for tool validation, Feel-fit accelerometer recorded the minutes of movement (MVPA) per day. The correlation of cumulative minutes MVPA measured by questionnaire and the objective measure resulted in a value of 0.809, indicating that GPAQ v.2 Thai version is in acceptable validity level to measure PA of Thai population.

### Data analysis

We employed a descriptive statistic such as frequency of distribution, central tendency (mean) and SD to explore the level, draw the trend and patterns of sufficient MVPA by survey year, age group and sex. Prevalence of sufficient MVPA is also presented by socio-demographic characteristics such as gender, age group, occupation, education, marital status, and area of residence (urban/rural). Considering its importance in differentiating PA, the presence of chronic disease (i.e. cancer, diabetes mellitus, cardiovascular diseases, heart disease, hypertension, stroke, high cholesterol, and kidney failure) that diagnosed by a medical doctor was also included in the analysis. Proportion of Thai population with sufficient MVPA in the last survey round (SPA2019) is also presented by domain (work-related, transportation and recreational) to describe PA differentials of Thai adults. Cumulative minutes of MVPA was also presented as additional information whenever necessary.

### Ethical approval

The protocol and data collection of SPAs were carried out in accordance with relevant guidelines and regulations. Participants of SPAs were informed of the objectives of the study, their rights to participate or withdraw at their convenience, and indicated their agreement by signing the informed consent. SPAs received ethical approval from the Institute for Population and Social Research of Mahidol University with annual updates: COA. N0. 2016–07-166 (SPA2016), COA. N0. 2017–06-152 (SPA2017), COA. No. 2018.06–192(SPA2018), COA. No. 2019/04–152 (SPA2019).

## Results

### Prevalence of sufficient MVPA

The proportion of sample was almost equal between genders and urban/rural residents in all SPA rounds. In average, Thai middle-aged adults (35–64) constituted about 60% of the panel, whereas young adults (18–34) and older adults (65+) made up of 25 and 15%, respectively. The majority of sample were married, attained primary education, and occupied in agriculture sector. Compare to baseline (SPA2012), the proportion of Thai adults with chronic disease tended to be lower in the most recent rounds (Supplementary Table 1).

**Fig. 1:**
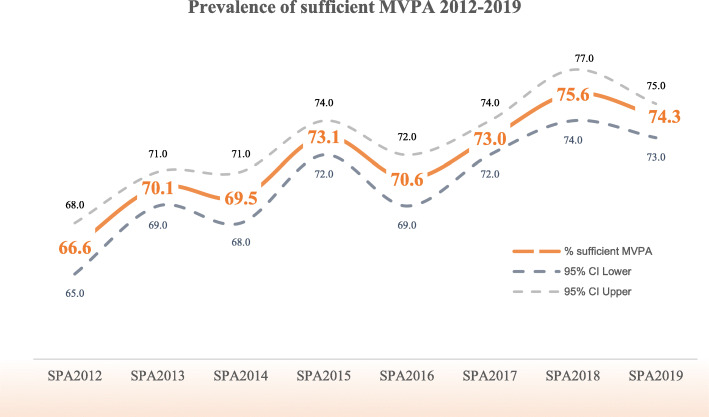
Prevalence of sufficient MVPA of Thai adults: 2012–2019

The prevalence of Thai adults who met WHO recommendations for sufficient PA tended to increase over time. At the beginning of Thailand’s SPA in 2012, 66.6% (CI 65–68) of Thai adults have met the guideline. The prevalence increased to 70.1 (CI 69–71), 69.5 (CI 68–71) and 73.1 (CI 72–74) percent during 2013–2015, and then slightly declined in 2016 (Fig. 1). The cumulative minutes of MVPA collected during a typical week showed a fluctuating trend; started at 705 mins (SD = 930, CI 681–730) in the baseline, increased to 828 mins (SD = 1003, CI 803–855) in SPA2013, declined during SPA2014–2016 then escalated in SPA2017–2018 before its fallen in SPA2019 (559 mins, SD = 682, CI 542–576) (Supplementary Table 2).

**Fig. 2:**
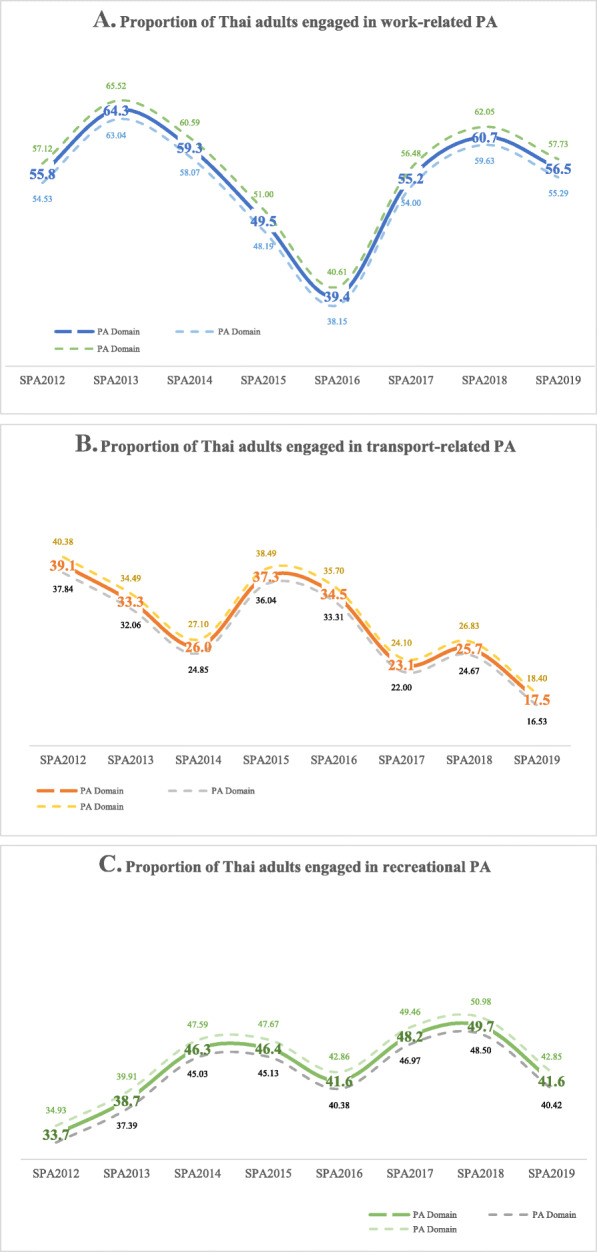
Proportion of Thai adults engaged in three domains of PA

Figure 2 presents the proportion of Thai adults engaged in three domains of PA. While the proportion of Thais who engaged in recreational PA was relatively stable between SPA2012 and SPA2018 (33.7 and 49.7%, respectively), there was a significant drop in the proportion of Thais who engaged in work-related PA from SPA2014 to SPA2016 before it rose back in the following years. While all three domains of PA showed a decline in the most recent (2019) round of the SPA, transport-related MVPA was lowest and decreased the most (Fig. 2). Work-related PA dominated the cumulative minutes of MVPA (x̄=493 mins, SD = 785) of total 631 min MVPA per week, followed by recreational PA (x̄=92 mins, SD = 179). Transport-related PA was consistently the least common type (x̄=42 mins, SD = 111) of PA throughout the 8 years of the SPA (Supplementary Table 3).

### Socio-demographic characteristics of Thai adults with sufficient MVPA

**Fig. 3:**
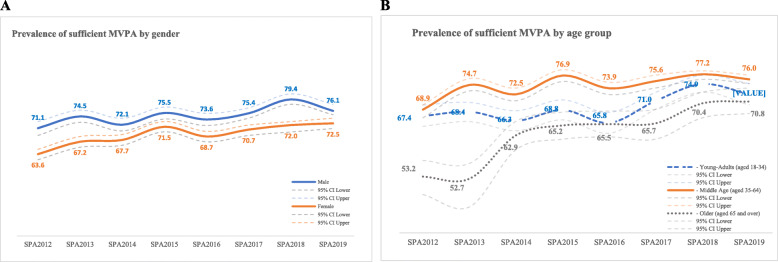
Prevalence of sufficient MVPA by gender and age group

Thai females are less physically active than their male counterparts. Throughout the eight rounds of the SPA, the prevalence of females with sufficient MVPA was consistently lower than males, ranging from 63.6 (CI 62–65) in SPA2012 to 72.5 (CI 71–74) percent in SPA2019. Among Thai males, there has been a fluctuating pattern with a tendency to increase over time, with the lowest prevalence of sufficient MVPA in SPA2012 (71.1%, CI 69–73), SPA2014 (72.1%, CI 70.3–74), and SPA2016 (73.6%, CI 71.9–75.4), whereas the highest prevalence was reported in SPA2018 (79.4%, CI 78–81) (Fig. 3a, Table 1).

**Table 1:** Prevalence of sufficient MVPA by socio-demographic characteristic of Thai adults

**Characteristics**	**SPA2012** **(*n*** **= 5648)**			**SPA2013** **(*n*** **= 5751)**			**SPA2014** **(*n*** **= 5840)**			**SPA2015** **(*n*** **= 5954)**		
	%	**95% CI**		%	**95% CI**		%	**95% CI**		%	**95% CI**	
		Lower	Upper		Lower	Upper		Lower	Upper		Lower	Upper
***Gender***												
Male	71.1	69.0	73.0	74.5	72.8	76.3	72.1	70.3	74.0	75.5	73.8	77.2
Female	63.6	62.0	65.0	67.2	65.6	68.7	67.7	66.2	69.3	71.5	70.1	73.0
***Age group (years)***												
Young adults (18–34)	67.4	65.0	70.0	68.4	66.1	70.6	66.3	64.0	68.7	68.8	66.5	71.2
Middle age (35–64)	68.9	67.0	70.0	74.7	73.3	76.2	72.5	71.0	74.0	76.9	45.5	78.3
Older adult (65+)	53.2	49.0	57.0	52.7	46.1	56.3	62.9	59.5	66.2	65.2	62.0	68.3
***Marital Status***												
Single	66.1	63.0	69.0	68.2	65.2	71.3	66.5	63.5	69.6	68.1	65.1	71.0
Married	68.3	67.0	70.0	72.3	70.9	73.6	71.2	69.8	72.6	75.2	73.9	76.5
Separated, widowed, divorced	57.0	53.0	61.0	59.5	55.7	63.2	63.7	60.2	67.2	67.8	64.5	71.1
***Education***												
Primary or lower	66.0	64.0	68.0	71.5	69.9	73.2	71.6	69.9	73.2	72.8	71.8	74.4
Secondary	68.1	66.0	70.0	71.3	69.2	73.4	67.9	65.8	70.0	73.7	71.7	75.7
Higher	65.5	63.0	68.0	63.3	60.2	66.3	66.6	63.7	69.5	73.1	70.4	75.7
***Occupation***												
Student	67.7	62.0	74.0	64.0	56.4	71.4	64.4	58.3	70.6	61.8	55.2	68.4
Private enterprise	67.0	64.0	70.0	71.7	69.0	74.3	71.9	69.3	74.5	74.0	71.4	76.5
Formal sector employee	64.8	62.0	68.0	62.3	59.1	65.5	65.2	62.1	68.3	72.8	69.9	75.8
Informal sector employee	72.9	70.0	76.0	78.3	75.8	80.8	75.0	72.3	77.6	77.4	75.1	79.8
Agriculture	76.1	74.0	78.0	84.1	82.1	86.0	76.1	73.8	78.4	79.3	77.1	81.4
Unemployed	54.4	52.0	57.0	52.9	50.0	55.7	59.0	56.2	61.8	62.9	60.1	65.7
***Have a chronic disease***												
Yes	66.7	65.0	68.0	67.7	65.6	69.8	n.a.	n.a.	n.a.	73.0	71.1	74.9
No	66.5	65.0	68.0	71.3	69.9	72.7	n.a.	n.a.	n.a.	73.2	71.8	74.6
***Area of residence***												
Urban	65.9	64.0	68.0	68.7	67.0	70.3	70.0	68.4	71.6	73.0	71.4	74.5
Rural	67.4	66.0	69.0	71.7	70.0	73.4	69.0	67.3	70.7	73.3	71.7	74.9
**Characteristics**	**SPA2016** **(*n*** **= 6074)**			**SPA2017** **(*n*** **= 6203)**			**SPA2018** **(*n*** **= 6252)**			**SPA2019** **(*n*** **= 6331)**		
	%	**95% CI**		%	**95% CI**		%	**95% CI**		%	**95% CI**	
		Lower	Upper		Lower	Upper		Lower	Upper		Lower	Upper
***Gender***												
Male	73.6	71.9	75.4	75.4	74.0	77.0	79.4	78.0	81.0	76.1	75.0	78.0
Female	68.7	67.2	70.2	70.7	69.0	72.0	72.0	70.0	73.0	72.5	71.0	74.0
***Age group (years)***												
Young adults (18–34)	65.8	63.4	68.3	71.0	69.0	73.0	74.9	73.0	77.0	72.5	70.0	75.0
Middle age (35–64)	73.9	72.5	75.3	75.6	74.0	77.0	77.2	76.0	78.0	76.0	75.0	77.0
Older adult (65+)	65.5	62.5	68.6	65.7	62.0	69.0	70.4	67.0	73.0	70.8	68.0	74.0
***Marital Status***												
Single	68.1	65.1	71.0	70.1	68.0	73.0	72.9	71.0	75.0	71.7	69.0	74.0
Married	72.2	70.8	73.5	74.6	73.0	76.0	76.7	75.0	78.0	75.8	74.0	77.0
Separated, widowed, divorced	66.0	62.7	69.2	69.4	66.0	73.0	74.5	71.0	77.0	70.8	68.0	74.0
***Education***												
Primary or lower	72.9	71.3	74.5	73.9	72.0	76.0	75.6	74.0	77.0	73.9	72.0	75.0
Secondary	69.0	67.0	71.0	73.3	72.0	75.0	77.4	76.0	79.0	74.3	72.0	76.0
Higher	67.4	64.6	70.3	70.5	68.0	73.0	72.3	70.0	75.0	75.1	73.0	77.0
***Occupation***												
Student	67.6	61.4	73.7	68.9	63.0	75.0	70.5	64.0	77.0	66.9	60.0	74.0
Private enterprise	67.7	65.0	70.4	71.4	69.0	74.0	73.8	71.0	76.0	75.7	73.0	78.0
Formal sector employee	67.0	64.1	70.0	70.2	67.0	73.0	73.2	70.0	76.0	75.5	73.0	78.0
Informal sector employee	72.9	70.3	75.5	79.3	77.0	81.0	79.7	77.0	82.0	74.5	72.0	77.0
Agriculture	80.8	78.7	82.8	84.1	82.0	86.0	87.5	86.0	89.0	83.1	81.0	85.0
Unemployed	63.2	60.5	65.9	62.5	60.0	65.0	66.4	64.0	69.0	66.1	64.0	68.0
***Have a chronic disease***												
Yes	72.8	70.7	74.9	73.5	72.0	74.0	75.8	74.0	78.0	75.8	74.0	78.0
No	69.8	68.5	71.2	72.8	70.0	75.0	75.5	74.0	77.0	73.6	72.0	75.0
***Area of residence***												
Urban	71.4	69.8	73.0	72.3	71.0	74.0	75.1	74.0	76.0	74.2	73.0	76.0
Rural	70.6	68.1	71.5	73.7	72.0	75.0	76.1	74.0	78.0	74.3	73.0	76.0

The prevalence of sufficient MVPA was highest among middle-aged adults (35–64 years), and the lowest among older adults (65+ years) (Fig. 3b). The proportion of young adults with sufficient MVPA was relatively stable from SPA2012 to SPA2016, but showed a slight increase afterward. Although the prevalence of sufficient MVPA was the lowest among other age groups, the proportion of older adults who met the recommended PA level tended to increase in the most recent rounds of the SPA. Detailed cumulative minutes of MVPA by gender and age group is available in the Supplementary Tables 4 and 5.

By marital status, the prevalence of sufficient MVPA was the highest among the married, and lowest among individuals who had separated from their spouse, or were divorced or widowed. The prevalence of single persons who met the guideline was relatively stable, between 66.1 (CI 63–69) to 68.1 (CI 65.1–71) percent in SPA2012–2016, and slightly increased to 70.1 (CI 68–73), 72.9 (CI 71–75) and 71.7 (CI 69–74) percent in SPA2017–2019, respectively (Table 1).

Compared to those who attained secondary education or higher, the prevalence of sufficient MVPA was consistently higher among individuals with primary education or lower, ranging from 66% (CI 64–68) in SPA2012 to 75.6% (CI 74–77) in SPA2018. The pattern of sufficient MVPA fluctuated among Thai adults with higher education, with the lowest prevalence (63.3%, CI 60.2–66.3) in SPA2013, and the highest (75.1%, CI 73–77) in SPA2019. Classified by occupation, the prevalence of sufficient MVPA was highest among individuals employed in agriculture (76.1–87.5%) and those working in the informal sector, and lowest among the unemployed and students (Table 1).

The prevalence of sufficient MVPA among urban and rural Thais was relatively similar. Despite minor fluctuations, the proportion of both urban and rural Thais who engaged in the recommended level of MVPA showed an increasing trend (Table 1). The gaps in the proportion of rural and urban residents who have sufficient MVPA narrowed in the most recent rounds of the SPA. The prevalence of sufficient MVPA was also similar between those with or without chronic disease. While a higher proportion of individuals without a chronic disease achieved the recommended level of MVPA during SPA2012–2014, the prevalence of sufficient MVPA was actually higher among those with chronic disease during SPA2015 onward (Table 1). Detailed cumulative minutes of MVPA by sociodemographic characteristics is available in the Supplementary Table 3.

Most Thai adults consistently engaged in work-related PA more than the other domains. Table 2 presents the proportion of Thai adults from SPA2019 who engaged in sufficient MVPA, classified by PA domain. Those who engage more in work-related PA are mostly male, middle-aged, married, attained secondary education, have no chronic disease, reside in a rural area, and work in the agricultural sector. Higher proportion of transport-related PA was found among females, older adults, those who separated/divorced/widowed, having primary education or less, and being unemployed. While both males and females had an equal proportion for recreational PA (41%), a higher proportion of older adults engaged in this domain than the other age groups. Urban residents, individuals with higher education, and those employed in the formal labor sector engaged more in recreational PA than the other domains (Table 2).

**Table 2:** Proportion of Thai adults who engaged in PA by domain in 2019

Characteristics	PA Domain								
	Work-related			Transportation			Recreational		
	%	95% CI		%	95% CI		%	95% CI	
		Lower	Upper		Lower	Upper		Lower	Upper
***Gender***									
Male	57.6	55.8	59.3	14.4	13.1	15.6	41.4	39.6	43.1
Female	55.5	53.8	57.2	20.3	18.9	21.7	41.9	40.1	43.5
***Age group (years)***									
Young adults (18–34)	57.8	55.3	60.1	13.6	11.9	15.1	37.4	35.1	39.8
Middle age (35–64)	58.7	57.1	60.3	17.6	16.3	18.7	42.1	40.4	43.6
Older adult (65+)	46.1	43.0	49.3	23.7	21.0	26.3	47.2	44.0	50.3
***Marital Status***									
Single	53.0	50.4	55.7	15.7	13.8	17.6	42.7	40.0	45.3
Married	58.9	57.4	60.4	17.3	16.1	18.4	40.7	39.2	42.2
Separated, widowed, divorced	49.9	46.4	53.3	21.5	18.6	24.2	44.6	41.1	47.9
***Education***									
Primary or lower	57.1	55.2	58.9	21.9	20.3	23.4	38.3	36.4	40.0
Secondary	59.3	57.2	61.3	14.6	13.1	16.0	39.5	37.5	41.5
Higher	50.2	47.5	53.0	13.2	11.3	15.0	52.6	49.8	55.3
***Occupation***									
Student	48.0	40.5	55.5	20.6	14.5	26.6	48.6	41.1	56.0
Private enterprise	55.8	53.2	58.3	13.3	11.5	15.0	44.3	41.7	46.8
Formal sector employee	51.9	48.9	54.9	14.2	12.1	16.3	50.9	47.9	53.9
Informal sector employee	64.5	61.7	67.2	16.3	14.2	18.4	31.9	29.3	34.6
Agriculture	75.9	73.3	78.5	20.6	18.1	23.0	33.7	30.8	36.6
Unemployed	40.9	38.3	43.4	22.5	20.3	24.7	45.1	42.4	47.6
***Have a chronic disease***									
Yes	54.4	52.1	56.5	18.3	16.5	20.0	47.9	45.6	50.1
No	57.5	56.0	58.9	17.1	16.0	18.2	38.8	37.4	40.3
***Area of residence***									
Urban	53.7	52.0	55.4	18.3	16.9	19.5	46.7	45.0	48.3
Rural	59.8	57.9	61.5	16.5	15.1	17.8	35.6	33.9	37.4

Formal sector employees include (1) civil servants, (2) politicians, (3) officers, (4) factory workers, and (5) retired civil servants. Informal sector employees include (1) freelancers and (2) professional athletes

## Discussion

Since the rapid epidemiological transition in the early 2000s, NCD have become the major cause of death of the Thai population, superseding infectious diseases [17, 18]. Among several NCD risk factors, physical inactivity contributed to 1.3% of total Disability-Adjusted Life Years (DALY) lost that could have been averted if individuals engaged in regular PA of at least 75-min vigorous intensity or 150-min combined moderate and vigorous intensity [13].

During 2001–2005, the Thai government implemented a “healthy life” campaign by promoting PA for more than 3 days per week [17], and has continuously developed a supportive, built-environment for PA in the past decade. The prevalence of Thai adults who met the WHO recommendation for sufficient PA showed an increasing trend, plausibly correlated to several healthy lifestyle campaigns during 2013–2015. These campaigns include the expansion of bike lanes to support the ‘Bike for Dad’ event that was held to honor King Rama IX’s birthday (observed as Thai Father’s Day), and ‘Bike for Mom’ to commemorate Queen Sirikit’s birthday, also celebrated as Thai Mother’s Day. The prevalence of sufficient MVPA then slightly decreased in 2016, perhaps reflecting a regression to the mean (after the campaign boom during 2013–2015).

As Thais recovered from the year-long mourning period with the passing of HM King Bhumibol Adulyadej in 2016, health promotion strategies shifted from royal-family branding to other public figures. The gradual increase in the prevalence of sufficient MVPA during 2017–2019 was possibly related to charity running events that gained widespread popularity among Thais. The nationwide campaign for marathons and ‘fun runs’ were implemented nationwide, and shifted the population’s perception toward running as competition to running for enjoyment. In 2017 alone, more than 900 running events [19, 20] were arranged by various organizations all over the country, and involved 2000–40,000 Thai runners in each event [21].

Thai females are less physically active than their male counterparts. This finding is consistent throughout the 9 years of the SPA, indicating there might be physical and socio-cultural barriers for females to engage in PA. The physiological construction of the female body comprises less skeletal frame and muscle mass than males [22]. That fact might reduce self-efficacy in performing PA for Thai females [23]. In addition, there is a socio-cultural expectation for Thai females to be calm and neat, and the normative Thai preference for fair skin may deter many women from outdoor, daytime PA [24]. It should be noted, however, that Thai women may actually accumulate more PA when all intensities and activities are considered [25], particularly when including household chores, which are characterized by long duration but low intensity, and typically performed by the women of the household.

The prevalence of sufficient MVPA was the highest among middle-aged adults (35–64 years). Many young adults are full-time students, and older adults have physical limitations to engage in MVPA. Thus, middle-aged adults have more opportunity to accumulate PA across all domains. As a typical developing nation from upper-middle income country, Thai adults collected work-related PA more than recreational and travel-related. Although the domain-specific cumulative minutes of Thai adults was generally lower compared to countries in the same group, the dominance of work-related towards the other domains was consistent with global patterns [26]. For adults who work in the formal labor sector, the workplace should be regarded as a venue for health-promoting exercise [27], e.g., by walking around the building, using stairs, and other light-to-medium PA in the office setting. Apart from recreational PA, collective active minutes could also be added from transport-related PA as part of the daily commute from home to workplace [28, 29]. Among adults employed in the agricultural or informal labor sectors, however, farming-related activity obviously dominates the overall pattern for PA. The prevalence of sufficient MVPA was highest among married individuals. As marital status implies social support, married individuals are more likely to receive support from their spouse compared to single persons or those who are separated/widowed/divorced. In this regard, being married also means having a steady partner to engage in regular PA with [30]. Previous studies found that a married person’s health behaviour is closely related to their spouse’s. Thus, the husband’s PA and/or sedentary behaviour might influence the wife’s, and vice versa [31].

Generally, there is no difference in the level of PA among Thai urban or rural residents. However, considering the PA domains, work-related PA dominated the cumulative minutes of MVPA per week, particularly among middle-aged adults who are occupied in the rural agricultural or informal sector. On the other hand, Thais who work in the formal labor sector in urban areas enjoy the privilege of recreational PA. That said, the lack of a difference in the overall PA by area of residence suggests that built-environment interventions provided by the Thai government may be having an impact in reducing rural-urban disparities. For example, the construction of village sports complexes (e.g., the Lankilaphat1 and 2 Project) have encouraged the rural community to engage more in PA. Similarly, in the urban and semi-urban setting, members of the local community are being encouraged to redesign or modify their environment to provide more opportunities for PA (e.g., the ‘healthy space model’). The provision of a sports complex and the expansion of the healthy space model have motivated and enabled community members to be more active through improved access to PA resources [32].

The higher prevalence of sufficient MVPA among those with primary education or less, and those employed in agriculture suggests that adult PA is closely related to the nature of the occupation and where they work. Agriculture obviously requires more physical movement than, say, an office job. Indeed, many of today’s workers in the formal sector are required to be engaged in screen media at a table and chair. Sitting in a stationary position throughout the work-day, with only little movement between spaces, has increasingly reduced the opportunity for occupational PA in that sector. The findings from this analysis indicate that work-place interventions in the formal labor sector are needed in order to increase the PA of the work force and reduce sedentary behavior. Short-break interventions with light-intensity PA have been shown to reduce sedentary time and, over a period of 12 months, result in a significant reduction in the BMI of those involved [33, 34].

The prevalence of sufficient MVPA was also similar between those with or without a chronic disease. That finding suggests that PA or other health-promoting exercise is a relevant prescription for prevention, treatment, and/or rehabilitation [35]. Studies have documented the value of PA as primary prevention towards NCD [36–38] and in improving natural immune response and stress relief [39–44]. As a form of treatment, evidence shows that people with a chronic disease are quite capable of performing aerobic PA, either in the clinical or home setting [35, 45].

The results of the study suggest that ISPAH’s (International Society for Physical Activity and Health) recommendation on eight investments that work for physical activity also could be applied to Thai context. As there is no single solution shown to be effective in improving PA, system based-approach that involves all components of community should be undertaken or enhanced, supported by public policies in national level that encourage and facilitate PA for all population. Since transport-related PA was constantly the lowest among the other domains, improvement in the active transport system such as safe infrastructure, connectivity, and an integrated urban and transport design could be an additional benefit for the Thai population in collecting more minutes of PA in their daily life. The provision of health education including through mass media should be continuously enhanced as a frequent reminder for Thais to engage in adequate PA, accompanied by sport and recreation opportunities for all population, particularly for the least active groups such as females. Workplace intervention should also be the focus in improving PA of Thai adults by encouraging their work force to engage in more occupational PA. This may entail modifying or redesigning the workplace to encourage -- or even require -- movement, as that should be beneficial in improving physical health, mental health, and the overall well-being of the employees.

As a longitudinal study with two-panel data, the findings of this study provide strong evidence for Thai government managers and policy makers of the prevalence and trends of sufficient PA of Thai adults over the past decade. The study provides a clear portrayal of the changes of PA over time, including identifying the gaps between the country’s target, policies being implemented, and the prevalence of sufficient PA as the outcome. As the SPA is conducted annually, PA prevalence could be a useful indicator for refining strategies in PA promotion in order to meet the national targets. Additionally, since the SPA collects data from a nationally-representative sample, the levels of PA prevalence could be regarded as reflective of the Thai population at large. The limitation of the study lies in the self-reported method of data collection through a structured questionnaire (GPAQ v.2), as that may drive under- or over-estimation of PA level due to respondents’ recall error or the desire to provide a culturally-appropriate response. However, the design of the SPA provides longitudinal data with identical sampling methods and representativeness, and those attributes may reduce or eliminate the errors.

## Conclusions

The prevalence of sufficient PA has fluctuated over time with a tendency to increase in the most recent years. Thai females are less physically active than males. The prevalence of sufficient MVPA was highest among middle-aged adults (35–64 years), married individuals, those with primary education or less, and those employed in agriculture. The proportion of sufficient MVPA was relatively similar among urban/rural dwellers or among Thais with/without a chronic disease. Work-related PA is the most common domain of Thai adults, but there is a lower prevalence of sufficient MVPA among workers in the formal labor sector than those in agriculture or the informal labor sector for this domain. These results implying further improvement in recreational physical activity is required. Workplace intervention should also be the focus in improving PA of Thai adults by encouraging their work force to engage in more occupational PA.

## Supplementary Information

**Additional file 1: Supplementary Table 1.** Sample characteristics.

**Additional file 2: Supplementary Table 2.** Cumulative minutes of MVPA of Thai adults 2012-2019 by socioeconomic characteristics.

**Additional file 3: Supplementary Table 3.** Cumulative minutes of MVPA by domains of PA.

**Additional file 4: Supplementary Table 4.** Cumulative minutes of MVPA by gender.

**Additional file 5: Supplementary Table 5.** Cumulative minutes of MVPA by age group.

## Data Availability

SPA data is available in TPAK repository, https://tpak.or.th/?p=4151

## Abbreviations

PA: Physical Activity
MVPA: Moderate-to-Vigorous Physical Activity
SPA: Surveillance on Physical Activity
GPAQ: Global Physical Activity Questionnaires
WHO: World Health Organization
NCD: Non-communicable Disease
DALY: Disability-Adjusted Life Year
