# Supplementary material for: Prevalence of sufficient MVPA among Thai adults: pooled panel data analysis from Thailand’s surveillance on physical activity 2012–2019
Source: BMC Public Health. 2021 Apr 7;21:665. doi: 10.1186/s12889-021-10736-6 (PMC8028057; doi:10.1186/s12889-021-10736-6)
Supplement: Supplementary file 1 — Additional file 1: Supplementary Table 1. Sample characteristics. [file 12889_2021_10736_MOESM1_ESM.docx]

## **Supplementary Table 1: Sample characteristics**

| **Characteristics** | **SPA12** | | **SPA13** | | **SPA14** | | **SPA15** | | **SPA16** | | **SPA17** | | **SPA18** | | **SPA19** | |
| --- | --- | --- | --- | --- | --- | --- | --- | --- | --- | --- | --- | --- | --- | --- | --- | --- |
|  | **n** | **%** | **n** | **%** | **n** | **%** | **n** | **%** | **n** | **%** | **n** | **%** | **n** | **%** | **n** | **%** |
| ***Gender*** |  |  |  |  |  |  |  |  |  |  |  |  |  |  |  |  |
| Male | 2,245 | 39.7 | 2,294 | 39.9 | 2,348 | 40.2 | 2,405 | 40.4 | 2,427 | 40.0 | 2,964 | 47.8 | 2,986 | 47.8 | 3,052 | 48.2 |
| Female | 3,403 | 60.3 | 3,457 | 60.1 | 3,492 | 59.8 | 3,549 | 59.6 | 3,647 | 60.0 | 3,239 | 52.2 | 3,266 | 52.2 | 3,279 | 51.8 |
| ***Age group (years)*** |  |  |  |  |  |  |  |  |  |  |  |  |  |  |  |  |
| Young adults (18-34) | 1,678 | 29.7 | 1,634 | 28.4 | 1,561 | 26.7 | 1,512 | 25.4 | 1,451 | 23.9 | 1,718 | 27.7 | 1,744 | 27.9 | 1,646 | 26.1 |
| Middle age (35-64) | 3,306 | 58.5 | 3,385 | 58.9 | 3,471 | 59.5 | 3,569 | 60.0 | 3,674 | 60.5 | 3,661 | 59.0 | 3,602 | 57.6 | 3,687 | 58.2 |
| Older adult (65+) | 664 | 11.8 | 732 | 12.7 | 808 | 13.8 | 872 | 14.6 | 949 | 15.6 | 824 | 13.3 | 906 | 14.5 | 984 | 15.5 |
| ***Marital Status*** |  |  |  |  |  |  |  |  |  |  |  |  |  |  |  |  |
| Single | 1,038 | 18.4 | 907 | 15.8 | 941 | 16.1 | 939 | 15.8 | 952 | 15.7 | 1,346 | 21.7 | 1,397 | 22.4 | 1,373 | 21.7 |
| Married | 3,966 | 70.2 | 4,173 | 72.6 | 4,161 | 71.3 | 4,241 | 71.2 | 4,281 | 70.5 | 4,085 | 65.9 | 4,099 | 65.6 | 4,146 | 36.5 |
| Separated, widowed, divorced | 644 | 11.4 | 671 | 11.7 | 738 | 12.6 | 774 | 13.0 | 840 | 13.8 | 772 | 12.4 | 752 | 12.0 | 811 | 12.8 |
| ***Education*** |  |  |  |  |  |  |  |  |  |  |  |  |  |  |  |  |
| Primary or lower | 2,869 | 50.8 | 2,962 | 51.5 | 2,918 | 50.0 | 2,976 | 50.0 | 3,000 | 49.4 | 2,666 | 43.0 | 2,678 | 42.8 | 2,731 | 43.1 |
| Secondary | 1,779 | 31.5 | 1,849 | 32.2 | 1,886 | 32.3 | 1,924 | 32.3 | 1,997 | 32.9 | 2,198 | 35.4 | 2,242 | 35.9 | 2,311 | 36.5 |
| Higher | 1,000 | 17.7 | 937 | 16.3 | 1,036 | 17.7 | 1,054 | 17.7 | 1,057 | 17.7 | 1,339 | 21.6 | 1,330 | 21.3 | 1,288 | 20.3 |
| ***Occupation*** |  |  |  |  |  |  |  |  |  |  |  |  |  |  |  |  |
| Student | 232 | 4.1 | 161 | 2.8 | 236 | 4.0 | 212 | 3.6 | 225 | 3.7 | 212 | 3.4 | 220 | 3.5 | 175 | 2.8 |
| Private enterprise | 1,070 | 18.9 | 1,120 | 19.5 | 1,149 | 19.7 | 1,156 | 19.4 | 1,140 | 18.8 | 1,306 | 21.1 | 1,366 | 21.9 | 1,451 | 22.9 |
| Formal sector employee | 890 | 15.8 | 886 | 15.4 | 912 | 15.6 | 895 | 15.0 | 973 | 16.0 | 1,034 | 16.6 | 1,126 | 18.0 | 1,059 | 16.7 |
| Informal sector employee | 940 | 16.6 | 1,038 | 18.1 | 1,054 | 18.1 | 1,175 | 19.7 | 1,111 | 18.3 | 1,263 | 20.4 | 1,214 | 19.4 | 1,191 | 18.8 |
| Agriculture | 1,182 | 20.9 | 1,333 | 23.2 | 1,333 | 22.8 | 1,364 | 23.0 | 139 | 23.0 | 1,055 | 17.0 | 1,064 | 17.0 | 1,032 | 16.3 |
| Unemployed | 1,334 | 23.6 | 1,212 | 21.1 | 1,156 | 19.8 | 1,152 | 19.3 | 1,226 | 20.2 | 1,332 | 21.5 | 1,261 | 20.2 | 1,422 | 22.5 |
| ***Have a chronic disease*** |  |  |  |  |  |  |  |  |  |  |  |  |  |  |  |  |
| Yes | 2,810 | 49.8 | 2,728 | 47.4 | n.a. | n.a. | 2,075 | 34.9 | 1,676 | 27.6 | 4,508 | 27.3 | 1,453 | 23.2 | 1,949 | 30.8 |
| No | 2,838 | 50.2 | 3,023 | 52.6 | n.a. | n.a. | 3,879 | 65.1 | 4,398 | 72.4 | 1,695 | 72.7 | 4,799 | 76.8 | 4,382 | 69.2 |
| ***Area of residence*** |  |  |  |  |  |  |  |  |  |  |  |  |  |  |  |  |
| Urban | 2,972 | 52.6 | 3,830 | 66.6 | 3,062 | 52.4 | 3,107 | 52.2 | 3,192 | 52.6 | 3,301 | 53.2 | 3,339 | 53.4 | 3,427 | 54.1 |
| Rural | 2,676 | 47.4 | 1,921 | 33.4 | 2,778 | 47.6 | 2,847 | 47.8 | 2,882 | 47.4 | 2,902 | 46.8 | 2,913 | 46.6 | 2,904 | 45.9 |
