# Supplementary material for: Prevalence of sufficient MVPA among Thai adults: pooled panel data analysis from Thailand’s surveillance on physical activity 2012–2019
Source: BMC Public Health. 2021 Apr 7;21:665. doi: 10.1186/s12889-021-10736-6 (PMC8028057; doi:10.1186/s12889-021-10736-6)
Supplement: Supplementary file 2 — Additional file 2: Supplementary Table 2. Cumulative minutes of MVPA of Thai adults 2012-2019 by socioeconomic characteristics. [file 12889_2021_10736_MOESM2_ESM.docx]

## **Supplementary Table 2: Cumulative minutes of MVPA of Thai adults 2012-2019 by socioeconomic characteristics**

| **Characteristics** | **SPA2012  (n=5,648)** | | | | **SPA2013  (n=5,751)** | | | | **SPA2014  (n=5,840)** | | | | **SPA2015  (n=5,954)** | | | |
| --- | --- | --- | --- | --- | --- | --- | --- | --- | --- | --- | --- | --- | --- | --- | --- | --- |
|  | Mean | S.D | **95% CI** | | Mean | S.D | **95% CI** | | Mean | S.D | **95% CI** | | Mean | S.D | **95% CI** | |
|  |  |  | Lower | Upper |  |  | Lower | Upper |  |  | Lower | Upper |  |  | Lower | Upper |
| ***Overall MVPA (minutes)*** | **705** | **930** | **681** | **730** | **828** | **1003** | **803** | **855** | **673** | **821** | **652** | **694** | **545** | **765** | **526** | **565** |
| ***Gender*** |  |  |  |  |  |  |  |  |  |  |  |  |  |  |  |  |
| Male | 791 | 969 | 751 | 832 | 926 | 1045 | 883 | 969 | 739 | 877 | 704 | 775 | 629 | 839 | 596 | 663 |
| Female | 648 | 898 | 618 | 679 | 764 | 968 | 732 | 797 | 628 | 778 | 603 | 654 | 488 | 705 | 465 | 512 |
| ***Age group (years)*** |  |  |  |  |  |  |  |  |  |  |  |  |  |  |  |  |
| Young adults (18-34) | 699 | 922 | 655 | 744 | 753 | 948 | 708 | 800 | 643 | 799 | 604 | 683 | 520 | 747 | 483 | 559 |
| Middle age (35-64) | 770 | 971 | 737 | 803 | 951 | 1061 | 915 | 987 | 743 | 860 | 715 | 772 | 605 | 812 | 579 | 632 |
| Older adult (65+) | 397 | 627 | 350 | 445 | 431 | 668 | 383 | 480 | 428 | 616 | 385 | 471 | 342 | 527 | 307 | 377 |
| ***Marital Status*** |  |  |  |  |  |  |  |  |  |  |  |  |  |  |  |  |
| Single | 595 | 804 | 550 | 645 | 712 | 912 | 653 | 772 | 584 | 765 | 536 | 634 | 470 | 673 | 427 | 514 |
| Married | 755 | 962 | 725 | 785 | 885 | 1031 | 854 | 916 | 723 | 852 | 698 | 749 | 586 | 801 | 562 | 610 |
| Separated, widowed, divorced | 574 | 891 | 506 | 644 | 636 | 899 | 569 | 705 | 502 | 667 | 454 | 550 | 415 | 641 | 370 | 461 |
| ***Education*** |  |  |  |  |  |  |  |  |  |  |  |  |  |  |  |  |
| Primary or lower | 761 | 992 | 725 | 797 | 315 | 1075 | 876 | 954 | 718 | 850 | 687 | 749 | 578 | 809 | 550 | 608 |
| Secondary | 709 | 918 | 667 | 752 | 835 | 982 | 790 | 880 | 695 | 844 | 657 | 733 | 565 | 783 | 531 | 601 |
| Higher | 538 | 724 | 493 | 583 | 545 | 716 | 500 | 592 | 507 | 662 | 467 | 548 | 415 | 566 | 381 | 450 |
| ***Occupation*** |  |  |  |  |  |  |  |  |  |  |  |  |  |  |  |  |
| Student | 400 | 482 | 338 | 463 | 370 | 491 | 294 | 447 | 414 | 525 | 347 | 481 | 307 | 428 | 249 | 365 |
| Private enterprise | 766 | 1041 | 704 | 828 | 803 | 914 | 750 | 857 | 687 | 792 | 642 | 733 | 550 | 722 | 508 | 592 |
| Formal sector employee | 692 | 963 | 629 | 756 | 605 | 846 | 549 | 661 | 560 | 760 | 511 | 609 | 424 | 619 | 384 | 465 |
| Informal sector employee | 954 | 1110 | 883 | 1025 | 1111 | 1147 | 1042 | 1182 | 811 | 900 | 757 | 865 | 695 | 904 | 644 | 748 |
| Agriculture | 892 | 954 | 838 | 947 | 1244 | 1170 | 1181 | 1307 | 945 | 981 | 892 | 998 | 761 | 924 | 712 | 810 |
| Unemployed | 376 | 507 | 350 | 404 | 378 | 536 | 348 | 408 | 362 | 450 | 336 | 388 | 270 | 394 | 248 | 30 |
| ***Have a chronic disease*** |  |  |  |  |  |  |  |  |  |  |  |  |  |  |  |  |
| Yes | 712 | 944 | 677 | 747 | 745 | 940 | 703 | 787 | n.a. | n.a. | n.a. | n.a. | 497 | 708 | 466 | 527 |
| No | 698 | 915 | 665 | 732 | 871 | 1030 | 838 | 904 | n.a. | n.a. | n.a. | n.a. | 571 | 793 | 547 | 597 |
| ***Area of residence*** |  |  |  |  |  |  |  |  |  |  |  |  |  |  |  |  |
| Urban | 668 | 918 | 635 | 701 | 802 | 1003 | 767 | 838 | 609 | 772 | 582 | 637 | 514 | 731 | 488 | 540 |
| Rural | 747 | 941 | 711 | 729 | 858 | 1002 | 820 | 896 | 743 | 868 | 711 | 775 | 580 | 799 | 551 | 609 |

**Supplementary Table 2 (contd.): Cumulative minutes of MVPA of Thai adults 2012-2019 by socioeconomic characteristics**

| **Characteristics** | **SPA2016  (n=6,074)** | | | | **SPA2017  (n=6,203)** | | | | **SPA2018  (n=6,252)** | | | | **SPA2019  (n=6,331)** | | | |
| --- | --- | --- | --- | --- | --- | --- | --- | --- | --- | --- | --- | --- | --- | --- | --- | --- |
|  | Mean | S.D | **95% CI** | | Mean | S.D | **95% CI** | | Mean | S.D | **95% CI** | | Mean | S.D | **95% CI** | |
|  |  |  | Lower | Upper |  |  | Lower | Upper |  |  | Lower | Upper |  |  | Lower | Upper |
| ***Overall MVPA (minutes)*** | **408** | **648** | **392** | **425** | **630** | **790** | **610** | **650** | **670** | **782** | **651** | **689** | **559** | **682** | **542** | **576** |
| ***Gender*** |  |  |  |  |  |  |  |  |  |  |  |  |  |  |  |  |
| Male | 492 | 714 | 464 | 521 | 723 | 842 | 693 | 754 | 751 | 819 | 722 | 780 | 614 | 705 | 590 | 640 |
| Female | 352 | 593 | 333 | 371 | 544 | 730 | 519 | 570 | 596 | 739 | 571 | 622 | 507 | 657 | 485 | 530 |
| ***Age group (years)*** |  |  |  |  |  |  |  |  |  |  |  |  |  |  |  |  |
| Young adults (18-34) | 368 | 527 | 339 | 398 | 603 | 749 | 568 | 639 | 668 | 782 | 631 | 705 | 561 | 683 | 528 | 594 |
| Middle age (35-64) | 459 | 700 | 437 | 482 | 683 | 837 | 656 | 711 | 713 | 812 | 686 | 740 | 593 | 702 | 570 | 616 |
| Older adult (65+) | 269 | 507 | 237 | 301 | 448 | 609 | 406 | 490 | 503 | 626 | 463 | 545 | 429 | 585 | 392 | 466 |
| ***Marital Status*** |  |  |  |  |  |  |  |  |  |  |  |  |  |  |  |  |
| Single | 354 | 534 | 320 | 388 | 543 | 691 | 506 | 580 | 575 | 687 | 540 | 612 | 499 | 612 | 467 | 532 |
| Married | 439 | 680 | 419 | 459 | 680 | 829 | 654 | 705 | 715 | 813 | 691 | 740 | 597 | 712 | 576 | 619 |
| Separated, widowed, divorced | 313 | 582 | 274 | 353 | 518 | 716 | 467 | 568 | 598 | 756 | 544 | 652 | 463 | 623 | 420 | 506 |
| ***Education*** |  |  |  |  |  |  |  |  |  |  |  |  |  |  |  |  |
| Primary or lower | 455 | 707 | 430 | 480 | 679 | 828 | 648 | 711 | 695 | 797 | 666 | 726 | 577 | 713 | 550 | 604 |
| Secondary | 410 | 651 | 382 | 439 | 673 | 829 | 639 | 708 | 739 | 827 | 705 | 774 | 586 | 699 | 557 | 614 |
| Higher | 274 | 410 | 250 | 299 | 460 | 605 | 428 | 493 | 502 | 639 | 467 | 536 | 474 | 572 | 443 | 505 |
| ***Occupation*** |  |  |  |  |  |  |  |  |  |  |  |  |  |  |  |  |
| Student | 277 | 406 | 224 | 331 | 363 | 401 | 309 | 417 | 394 | 425 | 338 | 451 | 329 | 355 | 277 | 382 |
| Private enterprise | 316 | 504 | 287 | 345 | 568 | 763 | 527 | 610 | 641 | 769 | 600 | 682 | 540 | 684 | 505 | 575 |
| Formal sector employee | 282 | 429 | 255 | 309 | 485 | 616 | 448 | 523 | 559 | 709 | 518 | 601 | 512 | 623 | 475 | 550 |
| Informal sector employee | 537 | 797 | 490 | 584 | 870 | 936 | 819 | 922 | 840 | 859 | 792 | 889 | 699 | 814 | 653 | 745 |
| Agriculture | 676 | 849 | 632 | 721 | 1003 | 937 | 946 | 1059 | 992 | 917 | 937 | 1048 | 815 | 791 | 767 | 864 |
| Unemployed | 195 | 320 | 177 | 213 | 322 | 450 | 298 | 346 | 412 | 536 | 382 | 442 | 339 | 403 | 319 | 361 |
| ***Have a chronic disease*** |  |  |  |  |  |  |  |  |  |  |  |  |  |  |  |  |
| Yes | 378 | 619 | 349 | 408 | 656 | 809 | 632 | 680 | 640 | 767 | 601 | 680 | 521 | 650 | 492 | 550 |
| No | 419 | 658 | 400 | 439 | 561 | 735 | 526 | 596 | 679 | 787 | 657 | 701 | 576 | 696 | 556 | 597 |
| ***Area of residence*** |  |  |  |  |  |  |  |  |  |  |  |  |  |  |  |  |
| Urban | 373 | 581 | 353 | 393 | 576 | 749 | 550 | 602 | 605 | 715 | 581 | 629 | 503 | 618 | 482 | 524 |
| Rural | 447 | 713 | 421 | 473 | 691 | 830 | 661 | 722 | 744 | 847 | 714 | 775 | 625 | 746 | 598 | 653 |
