# Supplementary material for: Prevalence of sufficient MVPA among Thai adults: pooled panel data analysis from Thailand’s surveillance on physical activity 2012–2019
Source: BMC Public Health. 2021 Apr 7;21:665. doi: 10.1186/s12889-021-10736-6 (PMC8028057; doi:10.1186/s12889-021-10736-6)
Supplement: Supplementary file 3 — Additional file 3: Supplementary Table 3. Cumulative minutes of MVPA by domains of PA. [file 12889_2021_10736_MOESM3_ESM.docx]

## **Supplementary Table 3: Cumulative minutes of MVPA by domains of PA**

|  | Work-related | | Transportation | | Recreational PA | | Total MVPA | |
| --- | --- | --- | --- | --- | --- | --- | --- | --- |
|  | Minutes | SD | Minutes | SD | Minutes | SD | Minutes | SD |
| SPA2012 | 564 | 922 | 65 | 136 | 75 | 174 | 705 | 930 |
| SPA2013 | 709 | 991 | 47 | 106 | 72 | 159 | 828 | 1003 |
| SPA2014 | 550 | 801 | 33 | 84 | 89 | 175 | 673 | 821 |
| SPA2015 | 403 | 732 | 54 | 116 | 88 | 176 | 545 | 765 |
| SPA2016 | 285 | 625 | 44 | 95 | 78 | 167 | 408 | 648 |
| SPA2017 | 488 | 769 | 31 | 94 | 110 | 205 | 630 | 790 |
| SPA2018 | 524 | 773 | 40 | 123 | 105 | 200 | 700 | 789 |
| SPA2019 | 417 | 665 | 21 | 133 | 120 | 174 | 559 | 682 |
