# Supplementary material for: Prevalence of sufficient MVPA among Thai adults: pooled panel data analysis from Thailand’s surveillance on physical activity 2012–2019
Source: BMC Public Health. 2021 Apr 7;21:665. doi: 10.1186/s12889-021-10736-6 (PMC8028057; doi:10.1186/s12889-021-10736-6)
Supplement: Supplementary file 4 — Additional file 4: Supplementary Table 4. Cumulative minutes of MVPA by gender. [file 12889_2021_10736_MOESM4_ESM.docx]

## **Supplementary Table 4: Cumulative minutes of MVPA by gender**

|  | Male | | Female | |
| --- | --- | --- | --- | --- |
|  | Minutes | SD | Minutes | SD |
| SPA2012 | 791 | 969 | 648 | 898 |
| SPA2013 | 925 | 1045 | 764 | 968 |
| SPA2014 | 739 | 877 | 628 | 778 |
| SPA2015 | 629 | 839 | 488 | 705 |
| SPA2016 | 492 | 714 | 352 | 593 |
| SPA2017 | 723 | 842 | 544 | 730 |
| SPA2018 | 750 | 819 | 596 | 739 |
| SPA2019 | 614 | 705 | 507 | 657 |
