# Supplementary material for: Prevalence of sufficient MVPA among Thai adults: pooled panel data analysis from Thailand’s surveillance on physical activity 2012–2019
Source: BMC Public Health. 2021 Apr 7;21:665. doi: 10.1186/s12889-021-10736-6 (PMC8028057; doi:10.1186/s12889-021-10736-6)
Supplement: Supplementary file 5 — Additional file 5: Supplementary Table 5. Cumulative minutes of MVPA by age group. [file 12889_2021_10736_MOESM5_ESM.docx]

## **Supplementary Table 5: Cumulative minutes of MVPA by age group**

|  | Young Adult (18-34) | | Middle age (35-64) | | Older Adult (65+) | |
| --- | --- | --- | --- | --- | --- | --- |
|  | Minutes | SD | Minutes | SD | Minutes | SD |
| SPA2012 | 699 | 922 | 770 | 971 | 397 | 627 |
| SPA2013 | 753 | 948 | 950 | 1061 | 431 | 668 |
| SPA2014 | 643 | 799 | 743 | 860 | 428 | 616 |
| SPA2015 | 520 | 747 | 605 | 812 | 342 | 527 |
| SPA2016 | 368 | 527 | 459 | 700 | 269 | 507 |
| SPA2017 | 603 | 749 | 683 | 837 | 448 | 609 |
| SPA2018 | 667 | 782 | 713 | 812 | 503 | 626 |
| SPA2019 | 561 | 683 | 593 | 702 | 429 | 585 |
